# Enhanced Perception for Autonomous Vehicles at Obstructed Intersections: An Implementation of Vehicle to Infrastructure (V2I) Collaboration

**DOI:** 10.3390/s24030936

**Published:** 2024-01-31

**Authors:** Yanghui Mo, Roshan Vijay, Raphael Rufus, Niels de Boer, Jungdae Kim, Minsang Yu

**Affiliations:** 1 Energy Research Institute, Nanyang Technological University, Singapore 637141, Singapore; yanghui.mo@ntu.edu.sg (Y.M.); rvijay@ntu.edu.sg (R.V.); raphael_luke@hotmail.com (R.R.); 2 Autonomous a2z, Anyang-si 14067, Republic of Korea; jdkim@autoa2z.co.kr (J.K.); minsang@autoa2z.co.kr (M.Y.)

**Keywords:** vehicle–infrastructure cooperative perception, roadside sensing system, collaborative autonomous driving, obstructed scenarios, V2X communications

## Abstract

In urban intersections, the sensory capabilities of autonomous vehicles (AVs) are often hindered by visual obstructions, posing significant challenges to their robust and safe operation. This paper presents an implementation study focused on enhancing the safety and robustness of Connected Automated Vehicles (CAVs) in scenarios with occluded visibility at urban intersections. A novel LiDAR Infrastructure System is established for roadside sensing, combined with Baidu Apollo’s Automated Driving System (ADS) and Cohda Wireless V2X communication hardware, and an integrated platform is established for roadside perception enhancement in autonomous driving. The field tests were conducted at the Singapore CETRAN (Centre of Excellence for Testing & Research of Autonomous Vehicles—NTU) autonomous vehicle test track, with the communication protocol adhering to SAE J2735 V2X communication standards. Communication latency and packet delivery ratio were analyzed as the evaluation metrics. The test results showed that the system can help CAV detect obstacles in advance under urban occluded scenarios.

## 1. Introduction

Autonomous vehicles (AVs) have the potential to revolutionize mobility but face significant challenges, particularly in complex urban environments. One of the primary challenges lies in the limitations of vehicle-based perception systems and onboard computing capabilities. The limitations in perception capabilities for individual autonomous vehicles become particularly evident in settings where visual obstructions, such as other vehicles or obstacles, restrict the field of view [1,2]. This restriction not only poses a safety risk but also complicates the task of achieving a comprehensive understanding of the vehicle’s environment. However, in a variety of real-world traffic scenarios, occlusions or limited perception distances are among the most commonly encountered challenges. For example, in tunnels with limited visibility [3,4]; in parking lots or garages where accidents are frequent and driver behavior is unpredictable [5]; in complex urban intersections with line-of-sight obstructions and high-traffic-density areas [6,7]; and on highways where there is insufficient long-range perception [8], the limitations of a vehicle’s own perception systems become apparent. To achieve safe and reliable autonomous driving in complex traffic conditions, addressing the issue of visual occlusions is imperative.

In this context, vehicle–infrastructure cooperative perception (CP) technology based on Vehicle-to-Everything (V2X), as a significant component of Cooperative Intelligent Transport Systems (C-ITSs), has garnered significant interest due to its potential to enhance the limited perception capabilities of individual vehicles. It facilitates the exchange of perception information between vehicles and other components of the traffic ecosystem, including roadside infrastructure [9]. By integrating additional sensing data from roadside infrastructure, it expands the situational awareness of vehicles, providing autonomous driving systems with a more comprehensive understanding of the environment. This can significantly mitigate challenges posed by occlusions or long-distance perception, thereby improving decision-making quality and enhancing road safety [10,11].

At present, some research studies have focused on the design of roadside perception systems and their application in different scenarios, as shown in [12,13,14,15,16,17,18,19]. Additionally, other studies have concentrated on the design of CP, data fusion, and testing applications, as demonstrated in [20,21,22,23,24,25,26,27]. Furthermore, there are studies dedicated to the evaluation of use cases for C-ITS collaborative perception applications, such as road safety and traffic efficiency [28,29,30], and the quantification of basic performance and functional requirements based on V2X communication performance metrics [31]. However, there are few studies that have specifically focused on the design and implementation of road cooperative perception, or CP, for augmenting autonomous driving applications in the context of occlusion scenarios.

Therefore, this paper will focus on field-testing research aimed at enhancing roadside perception for Connected Automated Vehicles (CAVs) using an integrated experimental platform based on the developed LiDAR Infrastructure System, which covers various scenarios involving blind-spot obstructions. Additionally, it will analyze communication evaluation metrics such as latency and packet loss rates to assess the V2X communication performance in the tests. This offers valuable insights for the advancement of vehicle–infrastructure cooperative perception in the future. Compared with the existing work, the main contributions of this paper are as follows:

- We successfully established an integrated experimental platform, combining the open-source software of the Baidu Apollo Autonomous Driving System 7.0 (ADS) (Baidu Inc, Beijing, China), Cohda Wireless V2X (Cohda Wireless, Wayville, Australia) communication equipment, and the Autonomous a2z company’s independently developed LiDAR Infrastructure System (Autonomous a2z, Anyang-si, Korea), setting the stage for vehicle–infrastructure cooperative perception experiments.
- We conducted a series of experiments in various obstructed intersection scenarios at the CETRAN (Centre of Excellence for Testing & Research of Autonomous Vehicles—NTU) autonomous vehicle test track, with the objective of examining how roadside perception enhancement assists vehicles in detecting objects in obstructed scenarios.
- We adhered to the SAE J2735 V2X standards [32] in the experiments, utilizing Dedicated Short-Range Communications (DSRC) methods. A detailed analysis of communication latency and packet delivery ratio data was performed, providing a basis for optimizing vehicular cooperative communication technologies.

The rest of this paper is structured as follows: Section 2 reviews related work. Section 3 describes the system design and its implementation. Section 4 details the experimental tests, including the experimental setup, occluded scenarios, and visualization. Section 5 presents and analyzes the V2X communication evaluation with experimental results. Finally, Section 6 concludes the paper and outlines future work.

## 2. Related Work

In autonomous driving, the perception system plays a fundamental and decisive role, not only enhancing road safety and reducing traffic congestion, but also improving overall transportation efficiency, thereby representing a revolutionary change in the automotive industry. However, in certain traffic scenarios, the intrinsic limitations of vehicle-based sensors necessitate assistance from roadside sensing technologies. This literature review explores the recent advancements in roadside perception and vehicle–infrastructure cooperation perception and the methodologies employed for experimental validation. Intelligent roadside perception involves the use of sensors and intelligent infrastructure facilities to sense the dynamic road environment and enhance road safety and efficiency. At present, several studies have explored this area from different perspectives.

In the design of intelligent roadside perception technology, recent research has primarily leveraged technologies such as cameras, radar, and LiDAR to realize roadside perception. For instance, Refs. [18,23] adopted data from smart roadside LiDAR systems, utilizing them as infrastructure devices to extend the perception range of vehicles. A comprehensive review of roadside LiDAR technology, presented in [33], underscored the significance of considering the impacts of adverse weather conditions and identified the challenges that remain open. In another development, Liu [34] engineered a smart roadside system employing roadside radar and V2I/P2I communication to tackle line-of-sight obstructions. Zhao [33] and Miucic [24] utilized cameras as roadside perception devices, enabling the sensing of real-time dynamic information about vehicles within their range. Furthermore, some studies have integrated cameras and LiDAR or radar in intelligent roadside units to enhance the awareness of Connected Automated Vehicles (CAVs) [12].

For various scenarios and objectives of roadside perception, most research focuses on expanding the range of vehicle perception and enhancing the awareness of pedestrians. Some studies have specifically targeted scenarios such as obstructed views and distant objects, while others have concentrated on particular segments of urban roads and highways. Zhang [25] conducted research aiming at the testing and application of roadside perception systems at urban roundabout intersections. Liu [15] focused on extending the vehicle’s perception range through multiple infrastructure sensors, especially for distant objects, validating the system with key performance indicators. Additionally, the study also examined the fusion of data from infrastructure sensors to broaden a vehicle’s perception range, especially for objects not directly visible to onboard sensors. Merdrignac [13] conducted simulation experiments on vehicle–road cooperative perception in crucial areas like intersections, aiming to verify and enhance safety in these critical zones. Jandial [16] investigated the site selection and precise calibration of smart roadside facilities in complex and hazardous scenarios.

With the rapid development of new-generation communication technologies, intelligent roadside perception systems are increasingly utilized in vehicle–roadside cooperative perception to enhance the perception capabilities of CAVs and traffic systems. Several researchers have designed systems that integrate information at the roadside, using it to acquire precise and comprehensive dynamic information about road segments. Zhao [35] introduced a road credibility map based on the Dempster–Shafer theory, aiming to integrate information from vehicles and infrastructure. Similarly, Arnold [21] integrated real-time cooperative perception and then broadcast this information to surrounding vehicles. Additionally, some studies have been primarily designed to utilize roadside perception systems as roadside perception enhancements for CAVs, achieving vehicular safety through V2I or V2V communications. Liu [34] developed a system that utilizes perception data from roadside radar to address line-of-sight obstructions for vehicles. Shi [18] employed LiDAR data from smart streetlights as an infrastructure device to expand the vehicle’s perception range.

Furthermore, some studies have conducted research on data fusion in roadside perception and vehicle–road cooperative perception. Duan [36] investigated data fusion from roadside RGB cameras and LiDAR, aiming to enhance detection accuracy at intersections and in complex scenarios. Gabb [23] similarly explored various fusion methods, such as track-level fusion and data association, combining data from infrastructure sensors and onboard sensors to improve perception in complex scenarios. Zhang [25] developed a road cooperative perception system using a deep learning approach to fuse data from multiple sensors, achieving high detection accuracy and low latency. Piazzoni [22] introduced a cooperative perception error model, specifically designed to address line-of-sight obstruction issues. These studies collectively demonstrate the evolving methods in sensor data integration, emphasizing its crucial role in advancing autonomous vehicle technologies.

In their practical test experiments, most studies utilized DSRC to address the challenge of effective V2I information transmission [12,20,21,25]. To further refine V2X message optimization, some research has also been conducted on enhancing communication algorithms. For instance, Tsukada [21] carried out simulation experiments under heavy-traffic conditions to assess a priority-based algorithm for message propagation. Additionally, Thandavarayan [14] developed an algorithm for optimizing message generation rules in collective perception, aiming to improve overall perception capabilities. These advancements highlight the critical role of communication technologies in facilitating efficient and reliable data exchange in autonomous driving systems.

In summary, with respect to roadside perception enhancement for AVs and vehicle–road cooperative perception, research has been conducted in various areas, with some studies verifying their methods’ performance through physical tests. However, most existing simulations and field studies have primarily concentrated on experiments in conventional scenarios or single-obstruction scenarios. This paper will demonstrate, through field tests, how the developed roadside perception system assists CAVs in detecting obstacles in advance under occluded scenarios at urban intersections. Then, it will evaluate the V2X communication performance in the experimental tests. Thus, we provide important references for the future development of vehicle–infrastructure collaborative perception.

## 3. System Design

The system developed integrates several cutting-edge technologies to realize roadside augmented perception capabilities for Autonomous Vehicles (AV) through Vehicle-to-Everything (V2X) communication. It is designed by integrating LiDAR Infrastructure System (LIS), Cohda MK5 (Cohda Wireless, Wayville, Australia) communication equipment as V2X communication devices, and Baidu Apollo (Apollo) Autonomous Driving (Baidu Inc, Beijing, China), commonly referred to as Apollo. Additionally, a crucial component of the system is the LIS-Apollo Bridge. This bridge is established using Python classes, which are generated from Protocol Buffers (ProtoBuf) messages associated with Apollo’s perception modules. The generated Python classes have member functions that enable serialization and deserialization of the data, which is useful when sending it over the network to and from the LIS.

### 3.1. LiDAR Infrastructure System

**Figure 1:**
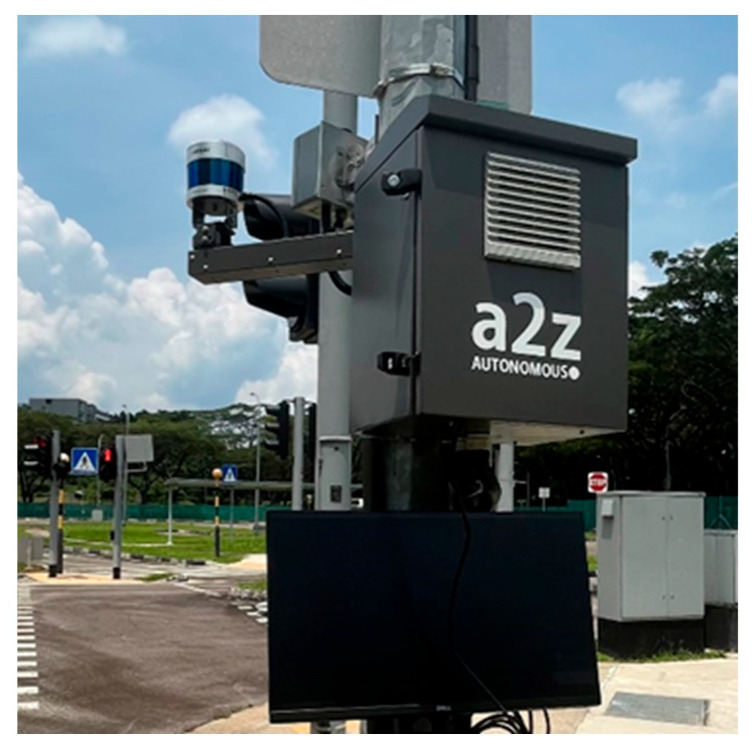
The developed LiDAR Infrastructure System setup.

The LiDAR Infrastructure System (LIS) (Autonomous a2z, Anyang-si, Korea) was developed by Autonomous a2z, which is an autonomous driving solution provider from South Korea. The LIS aims to enhance public road safety and facilitate traffic management as part of the Cooperative Intelligent Transport Systems (C-ITS). It provides a next-generation traffic service based on real-time data from a LiDAR sensor. As shown in Figure 1, the LIS consists of a road-side LiDAR (HESAI Pandar 40P) (Hesai Technology, Shanghai, China) for perception, a computer (VECOW SPC-5000) (Vecow, Taipei, Taiwan) with artificial intelligence software to integrate perceived information with a high-definition (HD) map, and a V2X Roadside Unit (RSU) radio (Cohda Wireless, Wayville, Australia). The LiDAR detects vehicles and pedestrians, the edge device processes the raw sensor data, and the RSU radio broadcasts the detection results to the CAVs. The vector mapping technology and localization algorithm in the HD mapping system enable rapid processing with minimal latency to the final terminal, such as a traffic control center or Onboard Units (OBUs) in vehicles.

The LIS can cover up to 200 m in distance and 360 degrees in angle at an intersection, and it is usually installed at intersections or heavy-traffic areas to detect real-time traffic situations. It provides various functionalities, including object detection, traffic signal prediction, and monitoring traffic violations and unexpected situations. Object detection involves identifying objects’ size, location, speed, and direction based on UTM or WGS84 coordinates. In actual applications, the LIS can provide information on traffic violations, such as illegal parking, unauthorized U-turns, speeding, and jaywalking, as well as unexpected situations like road construction or traffic accidents.

### 3.2. Vehicle

**Figure 2:**
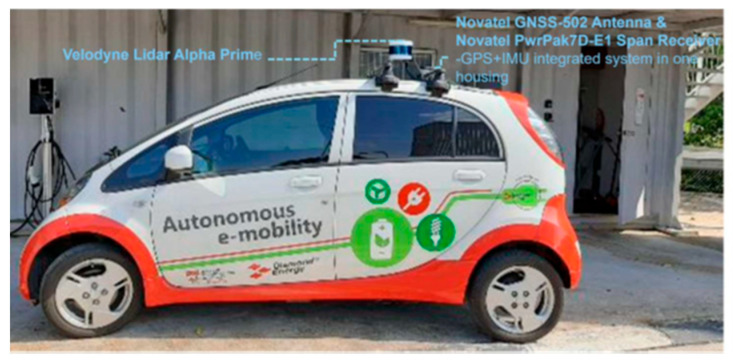
The vehicle platform with Baidu Apollo Automated Driving System.

Figure 2 shows an overview of the hardware configuration of the Connected Automated Vehicle (CAV) platform built by the NTU CETRAN group. The vehicle-based demonstrator is setup on a Mitsubishi iMiev (Mitsubishi Motors Corporation, Tokyo, Japan), and the primary components include an IPC processor that serves as the main processing unit, running the Baidu Apollo Automated Driving System (ADS) (Baidu, Beijing, China) on an Ubuntu 20.04 LTS (Canonical, London, United Kingdom). For sensing and localization, the vehicle is equipped with a Velodyne Alpha Prime (VLS-128) LiDAR sensor (Velodyne Lidar, San Jose, United States), which has 128 beams and provides a 360-degree horizontal and 40-degree vertical field of view (FOV), scanning the surroundings at a frame rate of up to 20 Hz. This LiDAR is calibrated to the vehicle’s local coordinate system using manual measurements with respect to the vehicle’s reference frame. Furthermore, the vehicle includes a Novatel PwrPak 7D-E1 GNSS (NovAtel, Calgary, Canada) receiver combined with an inertial measurement unit (IMU), offering precise localization and speed data. The GNSS system is augmented by a Novatel GNSS 502-Antenna (NovAtel, Calgary, Canada) and a Real-time Kinematic (RTK) correction service that uses Singapore Land Authority’s SiReNT service for improved accuracy.

For communication, the vehicle’s capabilities are enhanced with a Cohda Wireless MK5 OBU (Cohda Wireless, Wayville, Australia), enabling V2X communication, which is essential for the interconnected aspects of autonomous vehicular technology. The primary component of Apollo is CyberRT, an open-source, real-time, high-performance messaging framework [37]. It operates on a publish/subscribe messaging paradigm, like the Robot Operating System (ROS) framework. In this framework, subscribers or publishers must specify the CyberRT node and channel (similar to an ROS topic) that they intend to read from or write to. When the CAV receives messages through the OBU, the information about perceived objects is initially decoded from a binary ProtoBuf encoding. Subsequently, these data are transformed, along with their uncertainty, to the local frame of reference of the CAV.

### 3.3. Implementation of System

**Figure 3:**
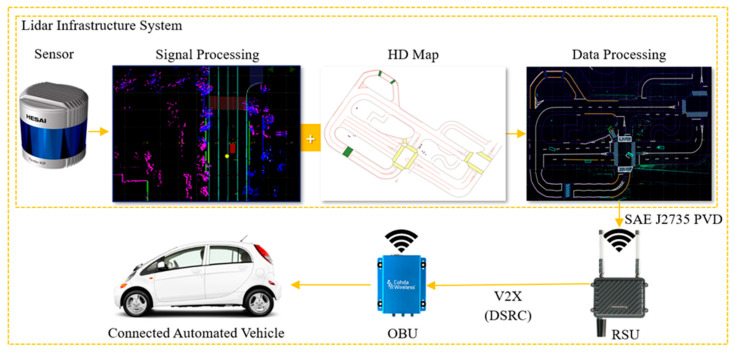
The flow of perception information from the LiDAR Infrastructure System to the Connected Automated Vehicle.

The LIS-based roadside perception enhancement for CAVs includes several steps, as shown in Figure 3. Initially, roadside LiDAR sensors capture and prepare the data. These data are then transmitted to the edge device of the LIS through an Ethernet connection. At the edge device, the sensed data undergo processing, which involves running perception algorithms for detection and tracking and integrating this perceived information with a high-definition (HD) map. Following this, the perception results are encoded into V2X messages. These encoded messages are then sent to the vehicle side via the RSU radio. On the vehicle side, the OBU radio decodes these messages and inputs the perception results into the Apollo ADS perception module.

During the implementation of the system, it employs Dedicated Short-Range Communications (DSRC) methods for transmitting information and package data, adhering to the SAE J2735 Protocol for V2X transmission [32], where the RSU broadcasts SAE J2735 PVD messages to the CAV through V2X devices. The LIS develops static and dynamic environment messages based on the ProtoBuf data format. The static environment messages, which include details like the location of the installed LIS, crosswalks, and crosswalk waiting areas, are programmed to be sent to the RSU once daily. On the other hand, the dynamic environment messages, conveying information such as the location, speed, and direction of obstacles, are programmed to be transmitted to the RSU every 100 milliseconds (ms).

## 4. Experimental Testing

### 4.1. Experimental Setup

**Figure 4:**
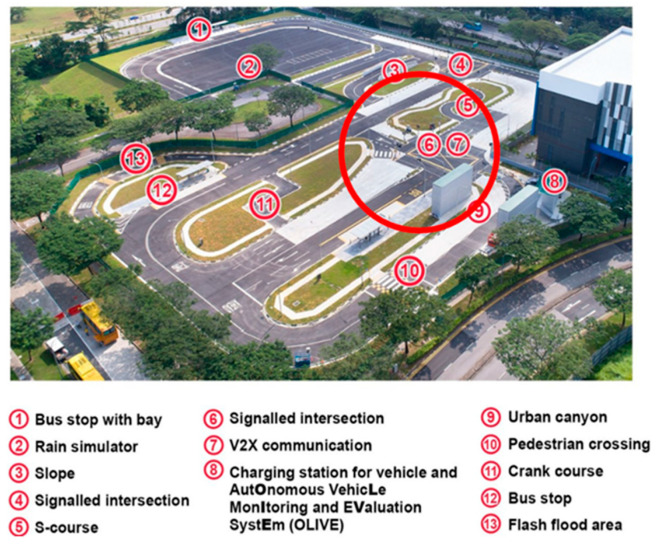
Experiment setup at the intersection of Singapore CETRAN (Centre of Excellence for Testing & Research of Autonomous Vehicles—NTU) autonomous vehicle test track.

The developed LiDAR Infrastructure System (LIS) and Connected Automated Vehicle (CAV) platforms, as previously described in Section 3, were used in the physical experiments. In the experiments, as labeled in Figure 4, the LIS was deployed on the lamppost of the intersection. The intersection (red circle) of the CETRAN AV test track was chosen mainly because it allows for the testing of a variety of common traffic scenarios, including vehicles making right turns and left turns, and scenarios involving pedestrians jaywalking. Additionally, a container was placed at this intersection to mimic the obstructing effect of high-rise buildings in urban settings, thereby making the experimental environment more akin to real urban traffic conditions.

Although the CAV could sense the surrounding environment using its built-in sensor, its perception sensor was not used for road user detection in the experiments presented in this paper. The CAV platform, therefore, practically functioned as a Connect Vehicle (CV) in the experiment and is referred to as the CV in the remainder of this section. The CV had to rely on the CP information from the LIS when interacting with pedestrians or other vehicles in the traffic environments. This highlights the benefits of using the CP service in CAV operations, especially in occluded scenarios.

### 4.2. Occlusion Scenarios and Visualization

The research involved conducting physical tests with the developed system through a full test track scenario and three types of occlusion scenarios. The full test track primarily aimed to test V2X communication between the LIS and the CV, measuring the communication performance as the CV moved along the entire test track and experienced signal blockages from buildings. The remaining three occluded scenarios included a left turn, a right turn, and pedestrian jaywalking, which could test the LIS’s ability to enhance the CV’s detection of hazards, particularly when there are visual obstructions. This is crucial for the CV’s timely hazard recognition and safe navigation, enhancing road safety and efficiency. This comprehensive approach aimed not only to test individual components of the system but also to evaluate the integrated performance of the LIS and CV in real-world traffic conditions, thus providing a more holistic understanding of the system’s capabilities and limitations.

**Figure 5:**
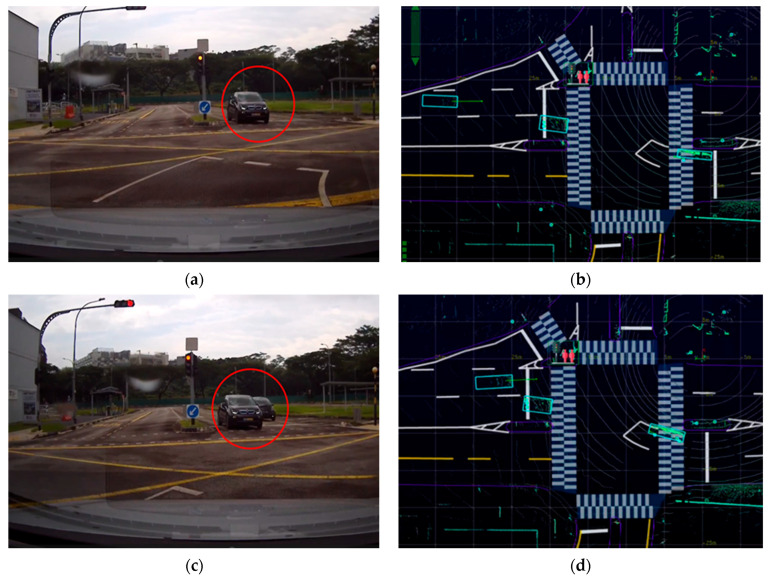
The road user detection and tracking in the right-turn scenario. In (**a**,**b**), the CV could “see” through the vehicle a visually occluded vehicle (labeled with red circles) with the help of the LIS. This occurred seconds before the CV drove closer and could observe by itself the previously occluded vehicle, as illustrated in (**c**,**d**).

**Figure 6:**
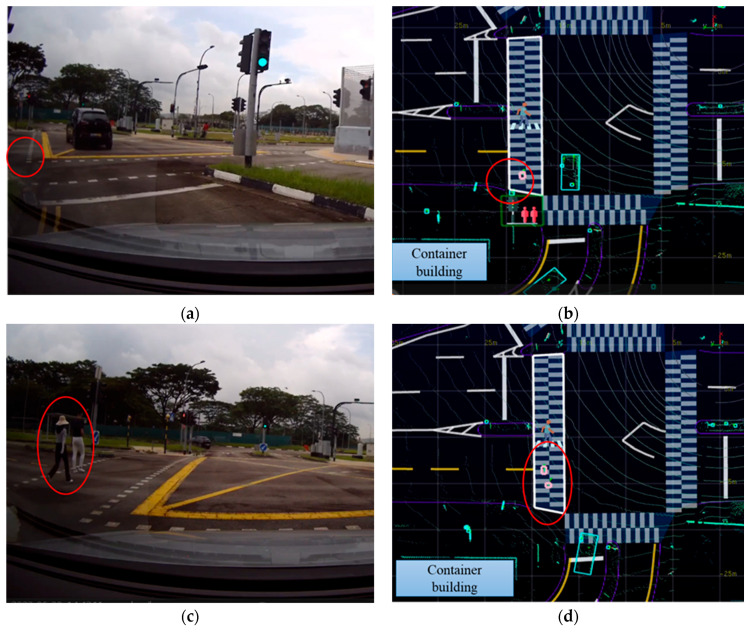
The road user detection and tracking in the left-turn scenario. In (**a**,**b**), the CV could “see” through the container visually occluded pedestrians (labeled with red circles) with the help of the LIS. This occurred seconds before the CV drove closer and could observe by itself the previously occluded pedestrians, as illustrated in (**c**,**d**).

**Figure 7:**
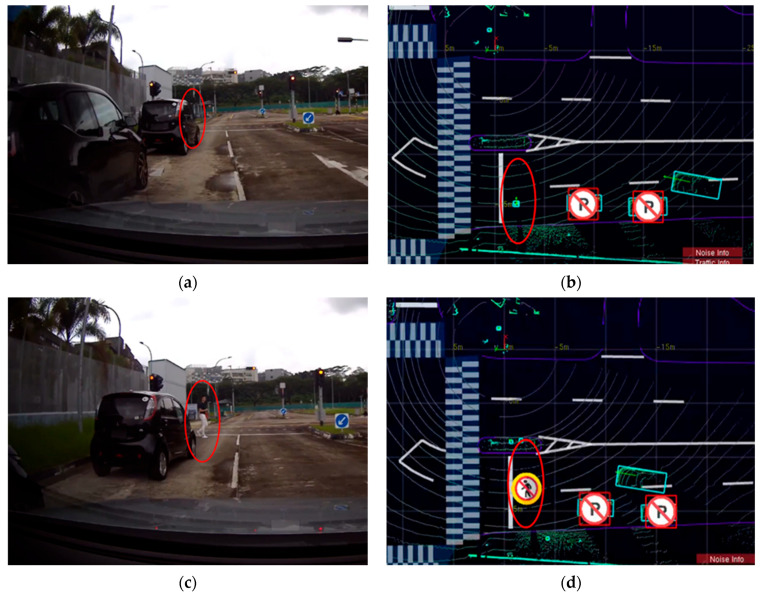
Road user detection and tracking in the pedestrian jaywalking scenario. In (**a**,**b**), the CV could “see” through the parking vehicles a visually occluded pedestrian (labelled with red circles) with the help of the LIS. This occurred seconds before the CV drove closer and could observe by itself the previously occluded pedestrian, as illustrated in (**c**,**d**).

In Figure 5, Figure 6 and Figure 7, the left column of each figure displays a sequence of images captured in the experiments by the front-right camera mounted on the CV at various time intervals. Meanwhile, the right column of each figure presents the corresponding road user tracking results as obtained from the LIS. Detailed descriptions and visualizations of the three traffic scenarios are presented below.

#### 4.2.1. The Right-Turn Scenario

As shown in Figure 5a,b, as the CV approached a right-turn maneuver, it obtained information about the ongoing traffic at the intersection from the LIS, which was positioned beyond the range of its onboard sensors. These data from the LIS enabled the CV to detect a straight-moving vehicle that was visually blocked by another vehicle turning right from the opposite direction. This detection was achieved seconds before the obstructed vehicle became visible to the CV’s own line of sight, as shown in Figure 5c,d. This early detection is critical for enhancing safety in both manual and autonomous driving scenarios. The experiment thus demonstrated the expanded sensing capabilities of the CV, made possible through the CP service, highlighting its significance in improving situational awareness and ensuring safer navigation.

#### 4.2.2. The Left-Turn Scenario

The left-turn scenario in this research was designed to simulate the “urban canyon” effect, a common urban environment wherein tall buildings at street corners can obscure pedestrians from the field of view of a CV. In Figure 6a,b, the CV demonstrated an advanced ability to “see” through these obstructions. With the assistance of sensing data received from the LIS, the CV could detect two pedestrians who were visually occluded by the corner building, as indicated by red circles in the figure. Notably, in Figure 6a,c, due to the limited field of view of the vehicle’s front-right camera, the container building located to the front left of the vehicle was not captured. However, it was detected by the LIS and can be seen in the bottom left corner of Figure 6b,d.

#### 4.2.3. The Pedestrian Jaywalking Scenario

In the pedestrian jaywalking scenario, the experiment simulated a situation wherein a pedestrian unexpectedly crossed the road while the CV’s line of sight was obstructed by vehicles parked at the roadside. This scenario represents a common urban challenge, where pedestrians may emerge from behind parked vehicles, creating a potential hazard for moving traffic. In Figure 7a,b, the CV demonstrated its enhanced perceptual capabilities in such a scenario. With the perception information received from the LIS, the CV could “see” and identify a pedestrian who was visually occluded by the parked vehicles well in advance. This early detection is critical for proactive safety measures, as it allows the CV to anticipate potential risks and adjust its maneuvers accordingly.

## 5. Experimental Evaluation

This section focuses on evaluating the V2X communication performance of the developed system platform between the LiDAR Infrastructure System (LIS) and the Connected Vehicle (CV). The evaluation encompasses the experimental tests conducted under the following scenarios: a full test track scenario and three types of occlusion scenario, as detailed in Section 4. During these tests, real-time data regarding changes in communication latency and packet delivery ratio (PDR) were collected as key metrics for this evaluation, as referenced in [28,29,38,39].

### 5.1. Latency

**Figure 8:**
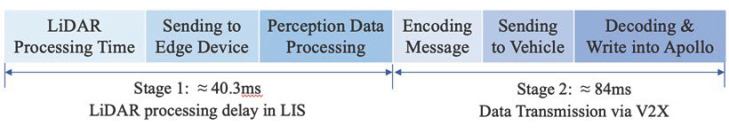
Delay measurement and breakdown in the field experiment.

Figure 8 presents the delay measurement and breakdown. The time delay experienced in the system primarily consisted of two key components:

1. Stage 1 was the LiDAR processing delay in the LIS, which included the time taken to obtain LiDAR data and transmit them to the LIS edge device, and the duration required for the perception algorithm to process the data on the edge device. This included the processing delay in the LIS pertaining to the clustering of the point cloud and the detection and localization of the objects. This value could not be directly derived from the system itself; it was measured by conducting tests.
2. Stage 2 was data transmission via V2X, which included the process of encoding the perception results, broadcasting them to the vehicle via DSRC, and then decoding and writing them into the vehicle’s Apollo system. This value was calculated by comparing the timestamps between when the perception message was published in the LIS and when the message was decoded and reaches the Apollo module.
  - To ensure the accurate synchronization of timestamps between various devices in the network, the Network Time Protocol (NTP) was initially employed in both the LIS and Apollo workstations. Subsequently, the timestamp differences were calculated between the perception message’s publication in the LIS and the message’s decoding and arrival at the Apollo module.

The total delay L is expressed as

L = L_lp_ + L_wr_ + L_rr_ + L_rw_ (1)

where L_lp_ represents the LiDAR processing time in the LIS, and L_wr_, L_rr_, and L_rw_ are parts of data transmission process, representing the transmission delays from the LIS workstation to the RSU, between the RSU and the OBU, and from the OBU to the Apollo workstation, respectively.

Table 1 shows the latency data of the full test track and occlusion scenarios in the field tests. The tests were conducted at the CETRAN AV test track, an enclosed site with a relatively small area. Given the observed minimal correlation between latency data variations and changes in distance range and road user count, Table 1 presents the average latency data for each scenario. The data illustrate that the total latency ranged from 110 to 130 ms. Even in the most challenging scenarios, the CV consistently received roadside perception messages from the LIS within 130 ms. Specifically, the Stage 1 latency for the LiDAR processing time (denoted as L_lp_) was about 40.3 ms. Additionally, the Stage 2 latency, which included the total time required for the data transmission processes L_wr_, L_rr_, and L_rw_, accumulated to around 84 ms. As a result, the overall average delay, represented by the symbol L, was around 124.3 ms.

### 5.2. Packet Delivery Ratio

**Figure 9:**
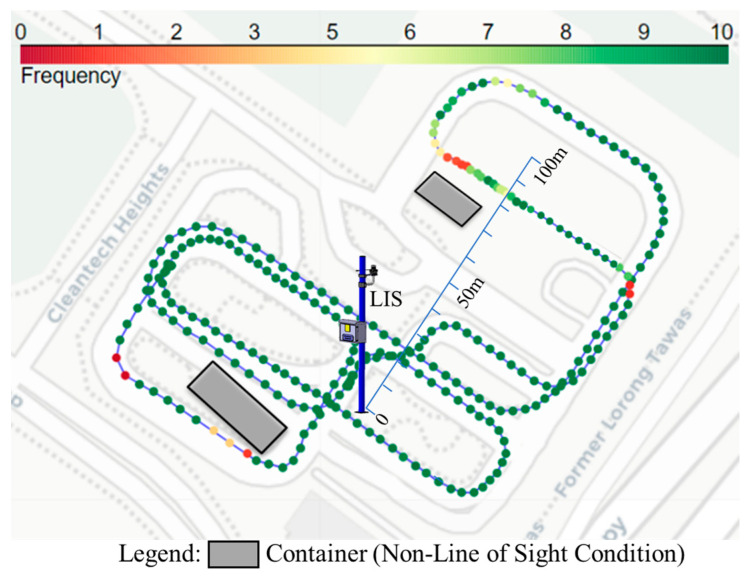
Packet delivery ratio in the field experiment.

In the experiments, the PDR was measured in one direction by comparing the Apollo logs of the sender and receiver. Notably, there were no packet losses in the Ethernet connection of the LIS system throughout all experiments. The frequency of point cloud measurement was 10 Hz. The PDR data, however, showed variations in building occlusions. In all occlusion scenarios, the CV was close to and remained visible to the LIS. Therefore, the PDR data presented in this section were primarily derived from the full test track data. Figure 9 shows the location of the LIS, the vehicle’s driving route, and the PDR data of the vehicle at different locations. The vehicle traveled along the blue line depicted in Figure 9 at an approximate speed of 15 km/h. The color variations of the circles in the figure indicate the PDR at specific locations, with areas lacking color indicating that no messages were received at those locations.

According to Figure 9, the physical occlusion, particularly by buildings, played a significant role in filtering messages and led to a sharp decline in PDR. This effect is especially evident in the areas to the lower left and directly above the LIS in the figure, where buildings were present. These obstructions resulted in decreased PDR values in the affected areas, thereby confirming the significant impact of structural barriers on signal propagation. It was also observed that in most cases, the PDR remained above 90% when the vehicle was within a 100-m radius of the LIS. Although a significant reduction in PDR can be noted directly above the center of the figure, these data were insufficient to conclude that the PDR gradually decreased as the distance from the LIS increased. As part of our future work, the authors will conduct further tests with the system outside of the enclosed test environment.

## 6. Conclusions

In summary, this paper presents the design, implementation, experimental testing, and V2X communication evaluation of a newly developed system for roadside perception enhancement in a CAV. The physical tests were conducted with the developed system at the CETRAN AV test track in Singapore. They demonstrated the system’s ability to assist the CAV in detecting in advance obstacles that would otherwise remain undetected by the vehicle at urban intersections with visual obstructions. Furthermore, the V2X communication evaluation results showed that the system could transmit roadside perception messages to CAVs in around 130 ms in the worst-case scenarios. It was also observed that the PDR decreased in the presence of building occlusions. This provides important reference information for the application of V2X perception enhancement in occlusion scenarios.

Future work is expected to also include field tests using the C-V2X 5G communication network for V2X transmission, aiming to compare the performance of both DSRC and C-V2X 5G across various scenarios. Additionally, we will incorporate more complex scenarios involving large-scale vehicular networks, such as prioritizing special vehicles at intersections and implementing coordinated green-wave traffic signals. In these experiments, the object data detected from the LIS via V2X were only visualized and compared with the images captured by the front camera of vehicle. However, future plans involve vehicle–roadside cooperative perception data for decision-making and planning processes.

## Figures and Tables

**Table 1:** Latency of full test track and occlusion scenarios in field tests.

	Full Test Track	Right Turn	Left Turn	Pedestrian Jaywalking	Overall Average
Stage 1 latency (ms)	40	41	43	37	40.3
Stage 2 latency (ms)	84	86	87	79	84
Total latency (ms)	124	127	130	116	124.3

## Data Availability

Data are contained within the article.
